# Supplementary material for: Data mining methodology for response to hypertension symptomology—application to COVID-19-related pharmacovigilance
Source: eLife. 2021 Nov 23;10:e70734. doi: 10.7554/eLife.70734 (PMC8754433; doi:10.7554/eLife.70734)
Supplement: Supplementary file 1. [file elife-70734-supp1.docx]

**Supplementary file 1.** 30 pulmonary ADEs.

| **30 pulmonary ADEs** | |
| --- | --- |
| Bacterial Lower Respiratory Tract Infections | Breathing Abnormalities |
| Bronchial Conditions NEC | Bronchospasm And Obstruction |
| Congenital Lower Respiratory Tract Disorders* | Coughing And Associated Symptoms |
| Fungal Lower Respiratory Tract Infections | Infectious Disorders Carrier |
| Lower Respiratory Tract Infections NEC | Lower Respiratory Tract Inflammatory And Immunologic Conditions |
| Lower Respiratory Tract Neoplasms | Lower Respiratory Tract Radiation Disorders* |
| Lower Respiratory Tract Signs And Symptoms | Occupational Parenchymal Lung Disorders |
| Parasitic Lower Respiratory Tract Infections* | Parenchymal Lung Disorders NEC |
| Pleural Conditions NEC | Pleural Infections And Inflammations |
| Pleural Neoplasms | Pneumothorax And Pleural Effusions NEC |
| Pulmonary Oedemas | Pulmonary Thrombotic And Embolic Conditions |
| Respiratory Failures (Excl Neonatal) | Respiratory Signs And Symptoms NEC |
| Respiratory Syncytial Viral Infections | Respiratory Tract Disorders NEC |
| Respiratory Tract Infections NEC | Respiratory Tract Neoplasms NEC* |
| Vascular Pulmonary Disorders NEC | Viral Lower Respiratory Tract Infections* |
| **5 unrelated pulmonary ADEs** | |
| Coronavirus Infections | Conditions Associated With Abnormal Gas Exchange |
| Neonatal Hypoxic Conditions | Newborn Respiratory Disorders Nec |
| Pulmonary Hypertensions |  |

* indicates 5 additionally deleted ADEs with no reports.
