# Supplementary material for: Data mining methodology for response to hypertension symptomology—application to COVID-19-related pharmacovigilance
Source: eLife. 2021 Nov 23;10:e70734. doi: 10.7554/eLife.70734 (PMC8754433; doi:10.7554/eLife.70734)
Supplement: Supplementary file 2. [file elife-70734-supp2.docx]

**Supplementary file 2.** Contribution of Pulmonary ADEs in 2D and 3D PCAs.

| **ADE in HLT codes** | **PC1 (%)** | **PC2 (%)** | **PC3 (%)** |
| --- | --- | --- | --- |
| Respiratory Failures (Excl Neonatal) | 8.02 | 2.36 | 0.00 |
| Respiratory Tract Disorders Nec | 7.81 | 1.53 | 0.01 |
| Bronchospasm and Obstruction | 7.80 | 0.92 | 0.94 |
| Parenchymal Lung Disorders Nec | 7.67 | 1.75 | 0.00 |
| Pulmonary Oedemas | 7.62 | 1.45 | 0.04 |
| Coughing and Associated Symptoms | 7.44 | 1.12 | 0.00 |
| Pneumothorax and Pleural Effusions Nec | 7.42 | 1.09 | 0.02 |
| Pulmonary Thrombotic and Embolic Conditions | 7.32 | 0.85 | 0.02 |
| Breathing Abnormalities | 7.15 | 0.62 | 0.01 |
| Lower Respiratory Tract Inflammatory and Immunologic Conditions | 6.89 | 0.32 | 0.03 |
| Lower Respiratory Tract Signs and Symptoms | 6.58 | 0.01 | 0.07 |
| Lower Respiratory Tract Infections Nec | 6.33 | 0.00 | 0.08 |
| Respiratory Signs and Symptoms Nec | 2.56 | 5.92 | 3.73 |
| Pleural Infections and Inflammations | 2.54 | 13.09 | 0.65 |
| Vascular Pulmonary Disorders Nec | 2.50 | 13.94 | 0.01 |
| Bronchial Conditions Nec | 2.45 | 10.83 | 0.64 |
| Respiratory Syncytial Viral Infections | 1.06 | 13.88 | 4.99 |
| Fungal Lower Respiratory Tract Infections | 0.41 | 10.39 | 63.19 |
| Pleural Conditions Nec | 0.36 | 14.84 | 0.87 |
| Lower Respiratory Tract Neoplasms | 0.04 | 2.44 | 11.18 |
| Infectious Disorders Carrier | 0.02 | 1.96 | 3.82 |
| Occupational Parenchymal Lung Disorders | 0.01 | 0.03 | 0.09 |
| Respiratory Tract Infections Nec | 0.00 | 0.01 | 9.33 |
| Pleural Neoplasms | 0.00 | 0.00 | 0.03 |
| Bacterial Lower Respiratory Tract Infections | 0.00 | 0.67 | 0.21 |
