# Supplementary material for: Data mining methodology for response to hypertension symptomology—application to COVID-19-related pharmacovigilance
Source: eLife. 2021 Nov 23;10:e70734. doi: 10.7554/eLife.70734 (PMC8754433; doi:10.7554/eLife.70734)
Supplement: Supplementary file 3. [file elife-70734-supp3.docx]

**Supplementary file 3.** Frequency of pulmonary ADEs when RR larger than two or the 5th quantile of EBGM, EB05, large than two.

| Drug | Pul. Count | Order by RR | Pul. Count | Order by EBGM |
| --- | --- | --- | --- | --- |
| ILOPROST | 10 | 2 | 13 | 1 |
| EPOPROSTENOL | 9 | 4 | 11 | 2 |
| SELEXIPAG | 10 | 3 | 11 | 3 |
| BERAPROST | 12 | 1 | 7 | 5 |
| CANDESARTAN | 3 | 33 | 5 | 9 |
| NIFEDIPINE | 5 | 15 | 5 | 10 |
| WARFARIN | 5 | 17 | 5 | 12 |
| BISOPROLOL | N/A | N/A | 4 | 13 |
| DILTIAZEM | 1 | 73 | 3 | 16 |
| HYDROCHLOROTHIAZIDE\RAMIPRIL | 5 | 13 | 3 | 17 |
| IMIDAPRIL | 5 | 14 | 3 | 18 |
| LERCANIDIPINE | 1 | 83 | 3 | 20 |
| NICARDIPINE | 4 | 27 | 3 | 21 |
| TELMISARTAN | 4 | 28 | 3 | 23 |
| TORSEMIDE | 5 | 16 | 3 | 24 |
| AZELNIDIPINE | 4 | 22 | 2 | 26 |
| AZILSARTAN KAMEDOXOMIL | 3 | 31 | 2 | 27 |
| BENDROFLUMETHIAZIDE | 3 | 32 | 2 | 28 |
| BENIDIPINE | 5 | 9 | 2 | 29 |
| CILNIDIPINE | 5 | 11 | 2 | 30 |
| HYDROCHLOROTHIAZIDE\VALSARTAN | 1 | 80 | 2 | 33 |
| METOPROLOL | 1 | 86 | 2 | 34 |
