# Supplementary material for: Data mining methodology for response to hypertension symptomology—application to COVID-19-related pharmacovigilance
Source: eLife. 2021 Nov 23;10:e70734. doi: 10.7554/eLife.70734 (PMC8754433; doi:10.7554/eLife.70734)
Supplement: Supplementary file 4. [file elife-70734-supp4.docx]

## Supplementary file 4. Arc Diagram

Here we introduced a new data visualization method, called arc diagram, which is accomplished by representing complex patterns of repetition in the analysis of ADE-drug combination data. The arc diagram improves over previous methods because they efficiently scale for a drug-ADE combination that detects many instances and signals of the same repeated sub-signals. **Figure 3-figure supplement 2** and its zoom level inset maps describe design and implementation related to parallel arc diagrams of ADE-drug combination and show how they may be applied to visualize such diverse data and compiled code. This arc diagram consists of selected drugs from GLASSO and pulmonary-related ADEs as the vertices, and arches denote the associations of either drug-drug or drug-ADE. Here we have highlighted three inset maps as examples of these highly correlated and complex interactions. The association regarding drug-drug and drug-ADE selected by GLASSO method for $EB05> 1$ was illustrated in an arc diagram (**Figure 3-figure supplement 2**). AHAs (macitentan, and bosentan) were found to have more pulmonary ADEs than other hypertensive drug classes. Two ATA drugs (epoprostenol and selexipag) showed more than 10 pulmonary ADEs. Given the clusters from GLASSO, all drugs except for group 1 had equal or less than five pulmonary ADEs. GLASSO group 1 consists of seven drugs from three ATC classes (from Table 3: AHAs, UAs, and ATAs). Combining RCM and arc diagram provides a more distinct way to demonstrate the association between drugs and their correlated pulmonary ADEs. The severity of pulmonary events associated with macitentan, bosentan, epoprostenol, selexipag, sildenafil, tadalafil, and beraprost was higher than the remaining drugs regarding six common ADEs in all of them. However, no more than two pulmonary issues were identical among drugs from the same GL Clusters 3, 4, 5, and 6, **Table 3**.
