# Supplementary material for: Data mining methodology for response to hypertension symptomology—application to COVID-19-related pharmacovigilance
Source: eLife. 2021 Nov 23;10:e70734. doi: 10.7554/eLife.70734 (PMC8754433; doi:10.7554/eLife.70734)
Supplement: Supplementary file 5. [file elife-70734-supp5.docx]

**Supplementary file 5.** Comparative analysis of drug and associated pulmonary ADEs in different GLASSO Clusters.

| Drug | Drug Class | | ADEs for EB05> 1 (n) * | GL Cluster |
| --- | --- | --- | --- | --- |
| ILOPROST | ATAs | | 2,4-13,16-17 (13) | 1 |
| EPOPROSTENOL | ATAs | | 1-2,4,7-13,16 (11) | 1 |
| SELEXIPAG | ATAs | | 1-2,4-5,7-8,10-13,16 (11) | 1 |
| BERAPROST | ATAs | | 1-2,4,10-13 (7) | 1 |
| WARFARIN | ATAs | | 1,4,8,10,13 (5) | 1 |
| CANDESARTAN | ARBs | | 1-3,6,15 (5) | 2 |
| NIFEDIPINE | CCBs | | 1-3,9,15 (5) | 2 |
| BISOPROLOL | BBAs | | 1-2,4,6 (4) | 3 |
| LERCANIDIPINE | CCBs | | 1,4,6 (3) | 3 |
| TORSEMIDE | TDAs | | 2-3,6 (3) | 3 |
| METOPROLOL | BBAs | | 2,6 (2) | 3 |
| DILTIAZEM | CCBs | | 2,5,9 (3) | 4 |
| HYDROCHLOROTHIAZIDE\VALSARTAN | COMBs | | 5,9 (2) | 4 |
| IMIDAPRIL | ACEs | | 1-3 (3) | 5 |
| TELMISARTAN | ARBs | | 1-3 (3) | 5 |
| AZELNIDIPINE | CCBs | | 1,3 (2) | 5 |
| AZILSARTAN KAMEDOXOMIL | ARBs | | 1,3 (2) | 5 |
| BENIDIPINE | CCBs | | 1-2 (2) | 5 |
| CILNIDIPINE | CCBs | | 1-2 (2) | 5 |
| HYDROCHLOROTHIAZIDE\RAMIPRIL | COMBs | | 2,7,14 (3) | 6 |
| NICARDIPINE | CCBs | | 3,7,14 (3) | 6 |
| BENDROFLUMETHIAZIDE | TDAs | | 3,14 (2) | 6 |
| * Below we have ADEs found for each drug: | |  | | |
| 1. PARENCHYMAL LUNG DISORDERS NEC | | 10. PARENCHYMAL LUNG DISORDERS NEC | | |
| 2. PNEUMOTHORAX AND PLEURAL EFFUSIONS NEC | | 11. PNEUMOTHORAX AND PLEURAL EFFUSIONS NEC | | |
| 3. LOWER RESPIRATORY TRACT INFLAMMATORY AND IMMUNOLOGIC CONDITIONS | | 12. LOWER RESPIRATORY TRACT INFLAMMATORY AND IMMUNOLOGIC CONDITIONS | | |
| 4. PULMONARY OEDEMAS | | 13. PULMONARY OEDEMAS | | |
| 5. BRONCHOSPASM AND OBSTRUCTION | | 14. BRONCHOSPASM AND OBSTRUCTION | | |
| 6. PULMONARY THROMBOTIC AND EMBOLIC CONDITIONS | | 15. PULMONARY THROMBOTIC AND EMBOLIC CONDITIONS | | |
| 7. RESPIRATORY TRACT DISORDERS NEC | | 16. RESPIRATORY TRACT DISORDERS NEC | | |
| 8. COUGHING AND ASSOCIATED SYMPTOMS | | 17. COUGHING AND ASSOCIATED SYMPTOMS | | |
| 9. LOWER RESPIRATORY TRACT INFECTIONS NEC | |  | | |
