# Supplementary material for: Data mining methodology for response to hypertension symptomology—application to COVID-19-related pharmacovigilance
Source: eLife. 2021 Nov 23;10:e70734. doi: 10.7554/eLife.70734 (PMC8754433; doi:10.7554/eLife.70734)
Supplement: Supplementary file 6. [file elife-70734-supp6.docx]

**Supplementary file 6.** Friedman test for drugs in ATC class and GLASSO class.

| ATC Class | p-value (44 drugs) | p-value (22 drugs) |
| --- | --- | --- |
| ACEIs | 0.229 | - |
| ARBs | **0.0056** | **0.0129** |
| ATAs | **< 0.001** | **0.007** |
| BBAs | **0.007** | 1 |
| CCBs | **0.00477** | 0.095 |
| COMBs | 0.369 | 0.819 |
| TDAs | 0.0552 | 0.108 |

| GL Cluster | p-value for 22 drugs |
| --- | --- |
| 1 | **0.00736**  (0.196, when excluding Warfarin) |
| 2 | 0.127 |
| 3 | 0.647 |
| 4 | 0.0736 |
| 5 | 0.0675 |
| 6 | 0.0840 |
