# Supplementary material for: Data mining methodology for response to hypertension symptomology—application to COVID-19-related pharmacovigilance
Source: eLife. 2021 Nov 23;10:e70734. doi: 10.7554/eLife.70734 (PMC8754433; doi:10.7554/eLife.70734)
Supplement: Supplementary file 7. — B. Multiple comparisons of different ATC classes excluding AHAs and UAs. [file elife-70734-supp7.docx]

**Supplementary file 7 A.** Multiple comparisons of different ATC classes together with the adjusted p-value using the rigorous paired Wilcoxon signed-rank test with Bonferroni correction.

| ATC Group | ATC Group | p-value | Adj. p-value | ATC Group | | ATC Group | | p-value | | Adj. p-value | |
| --- | --- | --- | --- | --- | --- | --- | --- | --- | --- | --- | --- |
| ACEIs | ARBs | 0.33 | 1 | ATAs | | BBAs | | **0.009** | | 0.341 | |
| ACEIs | ATAs | **0.021** | 0.763 | ATAs | | CCBs | | **0.006** | | 0.234 | |
| ACEIs | ATAs | **0.002** | 0.066 | ATAs | | COMBs | | **0.002** | | 0.084 | |
| ACEIs | BBAs | 0.267 | 1 | ATAs | | TDAs | | **0.015** | | 0.54 | |
| ACEIs | CCBs | 0.475 | 1 | ATAs | | UAs | | **0.006** | | 0.217 | |
| ACEIs | COMBs | 0.977 | 1 | BBAs | | CCBs | | 0.727 | | 1 | |
| ACEIs | TDAs | 1 | 1 | BBAs | | COMBs | | 0.433 | | 1 | |
| ACEIs | UAs | **0.024** | 0.86 | BBAs | | TDAs | | 0.401 | | 1 | |
| ARBs | AHAs | **0.043** | 1 | BBAs | | UAs | | **0.042** | | 1 | |
| ARBs | AHAs | **0.013** | 0.475 | CCBs | | COMBs | | 0.436 | | 1 | |
| ARBs | BBAs | 0.603 | 1 | CCBs | | TDAs | | 0.626 | | 1 | |
| ARBs | CCBs | 0.867 | 1 | CCBs | | UAs | | **0.027** | | 0.986 | |
| ARBs | COMBs | 0.495 | 1 | COMBs | | TDAs | | 0.698 | | 1 | |
| ARBs | TDAs | 0.455 | 1 | COMBs | | UAs | | **0.022** | | 0.785 | |
| ARBs | UAs | 0.066 | 1 | TDAs | | UAs | | 0.065 | | 1 | |
| AHAs | ATAs | **0.015** | 0.536 |  |  | |  | |  | |  |
| AHAs | BBAs | **0.034** | 1 |  |  | |  | |  | |  |
| AHAs | CCBs | **0.016** | 0.562 |  |  | |  | |  | |  |
| AHAs | COMBs | **0.009** | 0.338 |  |  | |  | |  | |  |
| AHAs | TDAs | **0.03** | 1 |  |  | |  | |  | |  |
| AHAs | UAs | 0.399 | 1 |  |  | |  | |  | |  |

**Supplementary file 7 B.** Multiple comparisons of different ATC classes excluding AHAs and UAs

| ATC Group | ATC Group | p-value | Adj. p |
| --- | --- | --- | --- |
| ACEIs | ARBs | 0.506 | 1 |
| ACEIs | ATAs | **0.005** | 0.102 |
| ACEIs | BBAs | 0.224 | 1 |
| ACEIs | CCBs | 0.354 | 1 |
| ACEIs | COMBs | 0.421 | 1 |
| ACEIs | TDAs | 0.344 | 1 |
| ARBs | ATAs | **0.017** | 0.357 |
| ARBs | BBAs | 0.494 | 1 |
| ARBs | CCBs | 0.449 | 1 |
| ARBs | COMBs | 0.745 | 1 |
| ARBs | TDAs | 0.399 | 1 |
| ATAs | BBAs | **0.011** | 0.237 |
| ATAs | CCBs | **0.014** | 0.284 |
| ATAs | COMBs | **0.011** | 0.235 |
| ATAs | TDAs | **0.016** | 0.342 |
| BBAs | CCBs | 0.966 | 1 |
| BBAs | COMBs | 0.495 | 1 |
| BBAs | TDAs | 0.702 | 1 |
| CCBs | COMBs | 0.704 | 1 |
| CCBs | TDAs | 0.637 | 1 |
| COMBs | TDAs | 0.723 | 1 |
