# Supplementary material for: Data mining methodology for response to hypertension symptomology—application to COVID-19-related pharmacovigilance
Source: eLife. 2021 Nov 23;10:e70734. doi: 10.7554/eLife.70734 (PMC8754433; doi:10.7554/eLife.70734)
Supplement: Supplementary file 8. — B. Multiple comparisons of drugs from GLASSO clusters and multiple comparisons of drugs from GLASSO clusters excluding Warfarin. [file elife-70734-supp8.docx]

**Supplementary file 8 A.** Multiple comparisons of drugs from GL Clusters and multiple comparisons of drugs from GL clusters excluding Tadalafil.

| GL Cluster | GL Cluster | Including Tadalafil | | Excluding Tadalafil | |
| --- | --- | --- | --- | --- | --- |
|  |  | p-value | Adj. p-value | p-value | Adj. p-value |
| 1 | 2 | **0.038** | 0.566 | **0.027** | 0.41 |
| 1 | 3 | **0.000579** | **0.009**** | **0.000355** | **0.005**** |
| 1 | 4 | **0.002** | **0.034*** | **0.000928** | **0.014*** |
| 1 | 5 | **0.004** | 0.056 | **0.002** | **0.024*** |
| 1 | 6 | **0.007** | 0.106 | **0.004** | 0.064 |
| 2 | 3 | 0.052 | 0.774 | 0.052 | 0.774 |
| 2 | 4 | 0.135 | 1 | 0.135 | 1 |
| 2 | 5 | **0.037** | 0.558 | **0.037** | 0.558 |
| 2 | 6 | 0.056 | 0.839 | 0.056 | 0.839 |
| 3 | 4 | 0.142 | 1 | 0.142 | 1 |
| 3 | 5 | 0.424 | 1 | 0.424 | 1 |
| 3 | 6 | 0.191 | 1 | 0.191 | 1 |
| 4 | 5 | 0.354 | 1 | 0.354 | 1 |
| 4 | 6 | 0.972 | 1 | 0.972 | 1 |
| 5 | 6 | 0.475 | 1 | 0.475 | 1 |

**Supplementary file 8 B.** Multiple comparisons of drugs from GLASSO clusters and multiple comparisons of drugs from GLASSO clusters excluding Warfarin.

| GL Cluster | GL Cluster | Including Warfarin | | Excluding Warfarin | |
| --- | --- | --- | --- | --- | --- |
|  |  | p-value | Adj. p-value | p-value | Adj. p-value |
| 1 | 2 | **0.027** | 0.41 | **0.012** | 0.186 |
| 1 | 3 | **0.008** | 0.116 | **0.004** | 0.058 |
| 1 | 4 | **0.027** | 0.41 | **0.012** | 0.186 |
| 1 | 5 | **0.006** | 0.095 | **0.003** | **0.047*** |
| 1 | 6 | **0.005** | 0.079 | **0.003** | **0.042*** |
| 2 | 3 | 0.205 | 1 | 0.205 | 1 |
| 2 | 4 | 0.559 | 1 | 0.559 | 1 |
| 2 | 5 | **0.05** | 0.747 | **0.05** | 0.747 |
| 2 | 6 | 0.154 | 1 | 0.154 | 1 |
| 3 | 4 | 0.884 | 1 | 0.884 | 1 |
| 3 | 5 | 0.204 | 1 | 0.204 | 1 |
| 3 | 6 | 0.639 | 1 | 0.639 | 1 |
| 4 | 5 | 0.176 | 1 | 0.176 | 1 |
| 4 | 6 | 0.537 | 1 | 0.537 | 1 |
| 5 | 6 | 0.516 | 1 | 0.516 | 1 |
