# Supplementary material for: Data mining methodology for response to hypertension symptomology—application to COVID-19-related pharmacovigilance
Source: eLife. 2021 Nov 23;10:e70734. doi: 10.7554/eLife.70734 (PMC8754433; doi:10.7554/eLife.70734)
Supplement: Supplementary file 9. [file elife-70734-supp9.docx]

**Supplementary file 9.** Dose distribution related to tadalafil and sildenafil and ADE.

| Tadalafil Dose | Frequency | Sildenafil Dose | Frequency |
| --- | --- | --- | --- |
| 40 | 5270 | 100 | 5997 |
| 20 | 5024 | 60 | 5775 |
| 5 | 3686 | 50 | 2913 |
| 1 | 1784 | 20 | 1647 |
| 10 | 1242 | 25 | 927 |
| 2.5 | 455 | 120 | 845 |
| 2 | 220 | 40 | 606 |
| 80 | 74 | 1 | 563 |
| 60 | 63 | 240 | 335 |
| 30 | 58 | 75 | 322 |
| 0.5 | 53 | 150 | 300 |
| 15 | 48 | 200 | 297 |
| 100 | 26 | 180 | 273 |
| 25 | 20 | 3 | 248 |
| 4 | 17 | 30 | 230 |
| 120 | 17 | 80 | 178 |
| 3 | 16 | 10 | 159 |
| 50 | 15 | 2 | 152 |
| 7.5 | 13 | 0.5 | 149 |
| 8 | 8 | 300 | 142 |
| 400 | 6 | 6 | 88 |
| 200 | 6 | 5 | 65 |
| 1.25 | 6 | 4 | 57 |
| 250 | 5 | 90 | 42 |
| 22 | 5 | 9 | 42 |
| 7 | 4 | 15 | 40 |
| 6 | 4 | 400 | 31 |
| 160 | 4 | 225 | 31 |
