# Supplementary figures and images for: Data mining methodology for response to hypertension symptomology—application to COVID-19-related pharmacovigilance
Source: eLife. 2021 Nov 23;10:e70734. doi: 10.7554/eLife.70734 (PMC8754433; doi:10.7554/eLife.70734)

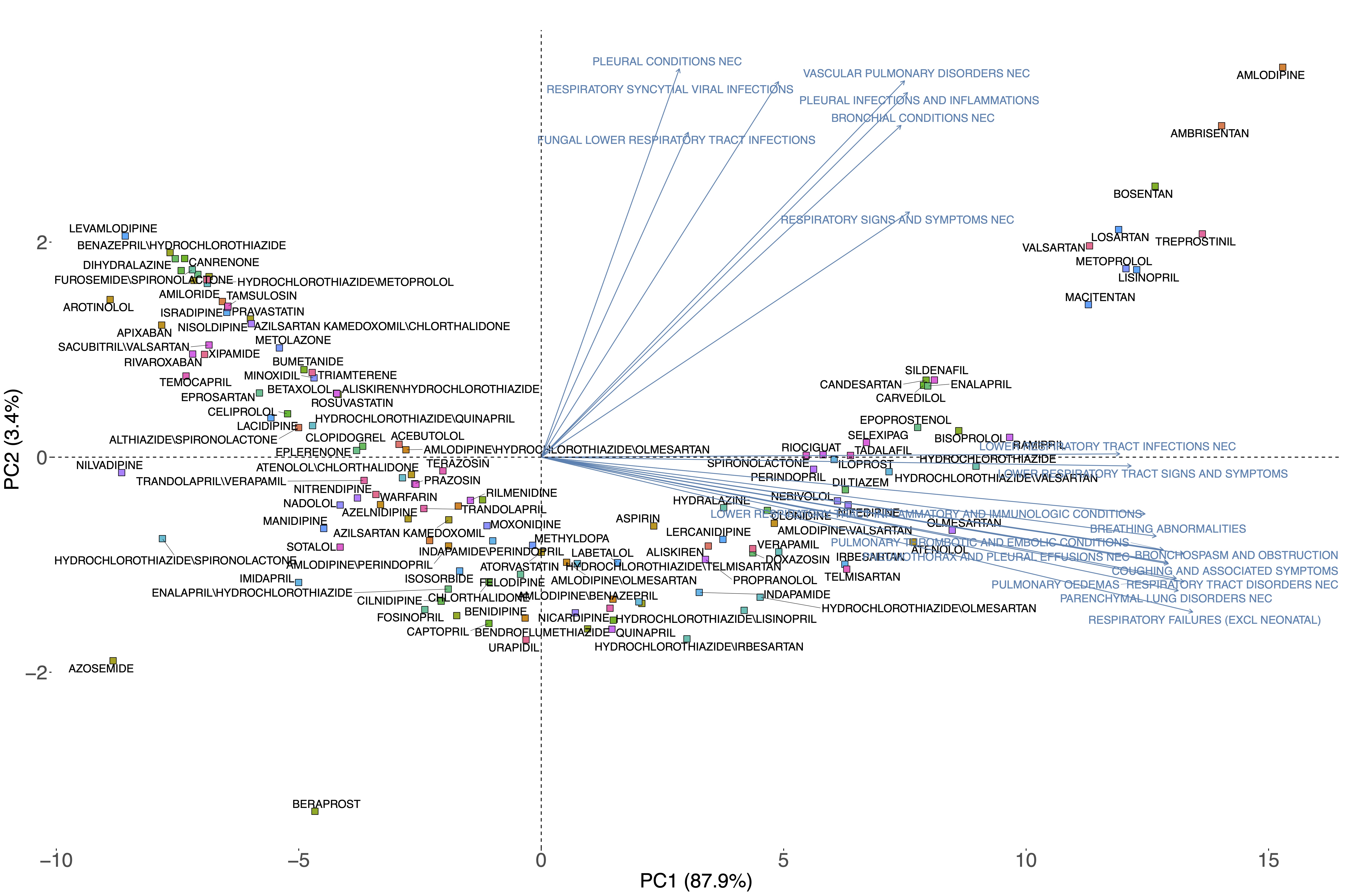

Supplement: Source data 1. [file elife-70734-supp10.zip › Source data/pca/Fig1-A.jpg]
